# An automated group-housed oral fentanyl self-administration method in mice

**DOI:** 10.1007/s00213-024-06528-6

**Published:** 2024-01-22

**Authors:** Noa Peretz-Rivlin, Idit Marsh-Yvgi, Yonatan Fatal, Anna Terem, Hagit Turm, Yavin Shaham, Ami Citri

**Affiliations:** 1 Edmond and Lily Safra Center for Brain Sciences, The Hebrew University of Jerusalem, Edmond J. Safra Campus, Givat Ram, 91904 Jerusalem, Israel; 2 Institute of Life Sciences, Hebrew University of Jerusalem, Edmond J. Safra Campus, Givat Ram, 91904 Jerusalem, Israel; 3 Behavioral Neuroscience Branch, IRP/NIDA/NIH, Baltimore, MD USA; 4 Program in Child and Brain Development, MaRS Centre, West Tower, Canadian Institute for Advanced Research, 661 University Ave, Suite 505, Toronto, ON M5G 1M1 Canada

**Keywords:** Oral fentanyl self-administration, HOMECAGE, Automated behavioral analysis, Group-housed, Social housing, Ethological, Naturalistic, Translationally-relevant

## Abstract

**Rationale and objectives:**

Social factors play a critical role in human drug addiction, and humans often consume drugs together with their peers. In contrast, in traditional animal models of addiction, rodents consume or self-administer the drug in their homecage or operant self-administration chambers while isolated from their peers. Here, we describe HOMECAGE (“Home-cage Observation and Measurement for Experimental Control and Analysis in a Group-housed Environment”), a translationally relevant method for studying oral opioid self-administration in mice. This setting reduces experimental confounds introduced by social isolation or interaction with the experimenter.

**Methods:**

We have developed HOMECAGE, a method in which mice are group-housed and individually monitored for their consumption of a drug vs. a reference liquid.

**Results:**

Mice in HOMECAGE preserve naturalistic aspects of behavior, including social interactions and circadian activity. The mice showed a preference for fentanyl and escalated their fentanyl intake over time. Mice preferred to consume fentanyl in bouts during the dark cycle. Mice entrained to the reinforcement schedule of the task, optimizing their pokes to obtain fentanyl rewards, and maintained responding for fentanyl under a progressive ratio schedule. HOMECAGE also enabled the detection of cage-specific and individual-specific behavior patterns and allowed the identification of differences in fentanyl consumption between co-housed control and experimental mice.

**Conclusions:**

HOMECAGE serves as a valuable procedure for translationally relevant studies on oral opioid intake under conditions that more closely mimic the human condition. The method enables naturalistic investigation of factors contributing to opioid addiction-related behaviors and can be used to identify novel treatments.

## Introduction

Social factors and the context of the drug intake environment play a critical role in human drug addiction, and humans often consume drugs together with their peers (Badiani et al. 2011; Heilig et al. 2016; Knight and Simpson 1996; Valente et al. 2007). In contrast, in traditional animal models of drug addiction, rodents typically consume or self-administer the drug in homecage or operant self-administration chambers while isolated from their peers (Ahmed 2010; Schuster and Thompson 1969). Laboratory animals tested outside their homecage are also exposed to confounds related to repeated interactions with the experimenter, which could impact their behavior (de Abreu and Kalueff 2021; Neff 2018).

The misuse of prescription opioids has resulted in a new population of people who suffer from opioid addiction who were initially introduced to opioid drugs during medical treatment (Howard et al. 2023; Sanger et al. 2020). Patients who misuse prescription fentanyl are more likely to misuse illicit fentanyl and other addictive drugs (Schepis et al. 2019). Prescription fentanyl can be administered through different routes, including the oral route via pills (Armenian et al. 2018; Lötsch et al. 2013). Additionally, illicit opioids, including fentanyl, are often distributed as pills (Armenian et al. 2018; Daniulaityte et al. 2022; Han et al. 2019; Sutter et al. 2017). Patients who seek non-fentanyl opiates on the street will likely consume fentanyl as well, since those pills are often blended with fentanyl due to its potency and cheaper production cost (Armenian et al. 2018; Daniulaityte et al. 2022; Han et al. 2019; Sutter et al. 2017). Thus, many people who are addicted to opioid drugs consume fentanyl orally and do so throughout the day. In this paper, we describe a fully automatic procedure that mimics the human condition of voluntary oral opioid self-administration in group-housed mice. In this procedure, as in the human condition, subjects are allowed to socially interact and voluntarily consume oral fentanyl 24 h per day.

**Fig. 1:**
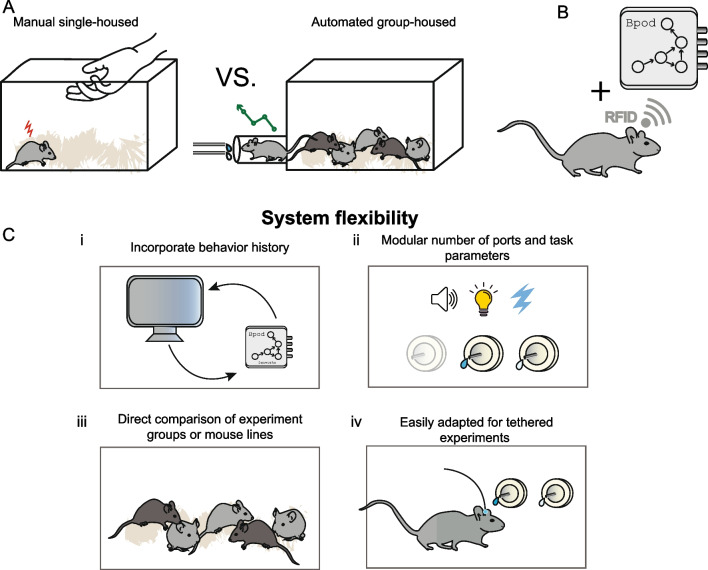
Overview of HOMECAGE, an automated group-housed oral self-administration procedure. **A** Most rodent studies of drug consumption or self-administration are performed on single-housed rodents, involving manual training by the experimenter. In this manuscript, we describe the HOMECAGE procedure that allows automated, individually tailored group-housed training. **B** HOMECAGE is based on the integration of an RFID chip for individual identification of participating subjects with the Sanworks Bpod state-machine, in the context of a homecage housing multiple mice. **C** The flexibility of this system affords multiple benefits, including (i) the capacity to tailor the individual task, online, according to recent behavioral history; (ii) the comparison of experimental groups that are co-housed; (iii) the possibility to tailor the task parameters and the number of ports; and (iv) the adaptation to tethered settings for optical recordings and/or manipulations

In the “HOMECAGE” (Home-cage Observation and Measurement for Experimental Control and Analysis in a Group-housed Environment) procedure, group-housed mice are individually and continuously monitored for their consumption of fentanyl vs a reference liquid (water or quinine-adulterated water) (Terem et al. 2023). The system maintains precise tracking of each individual mouse. This is important not only for documentation and analysis but also for individualized customization of experimental parameters. By integrating radio-frequency identifiers (RFIDs) into the experimental setup, the performance of each individual mouse can be efficiently monitored, and the requirements to obtain fentanyl reward can be flexibly adjusted according to each mouse’s behavioral history or experimental plan (Fig. 1B, C(i)). As mice may exhibit individual differences in the dynamics with which they escalate fentanyl consumption, the HOMECAGE setup can also be adjusted to the individual pace of each experimental subject. Specifically, the capability to continuously monitor the activity of individual mice and dynamically update task parameters enables the implementation of individualized progressive ratio protocols (see below). Additionally, the individual monitoring of the subjects allows for a direct comparison between experimental groups, while they are co-housed and experience the same conditions (Fig. 1C (ii)). Furthermore, this continuous tracking of individual mice enables the extraction of rich descriptions of patterns of drug consumption rather than simply quantifying daily drug consumption or preference. The HOMECAGE setup is highly flexible, allowing for the simple addition or removal of reward ports or sensory stimuli for different experimental requirements (Fig. 1C (iii)). The procedure can also be used with minimal adjustments for studying the consumption of isolated mice if the experimental setup requires it (Fig. 1C (iv)). Recently, the utility of the HOMECAGE setup was exemplified in an investigation implicating the claustrum in control of oral fentanyl self-administration (Terem et al. 2023).

## Materials and methods

### Animals

Mice described in the study were C57BL/6 inbred male mice purchased from Harlan Laboratories, Jerusalem. All mice were maintained on a 12-h light–dark cycle in a specific pathogen-free (SPF) animal facility with free access to food. All experimental procedures, handling, surgeries, and care of laboratory animals used in this study were approved by the Hebrew University Institutional Animal Care and Use Committee (IACUC).

### Surgery

Experimental manipulations of mice have been described in detail in Terem et al. (2023). Of relevance to the protocol described herein, an RFID chip (ID-20LA, ID Innovations) was implanted subcutaneously, between the shoulder blades of the mice, using a dedicated needle. After injection of the RFID tag, it was pushed lightly forward to lodge it more securely under the skin and ensure it will not be accidentally ejected. In the experiments described, RFID tag injection was performed during stereotactic surgery for virus injection. However, we have also experienced success with RFID implantation by applying transient anesthesia with reduced isoflurane doses. Mice were then disconnected from the anesthesia and were administered with subcutaneous saline injection (100 µl) for hydration and an IP injection of the analgesic Rimadyl (Norbrook, 5 mg/kg) as they recovered under gentle heating.

### Fentanyl

Fentanyl for oral consumption was obtained from Shaare Zedek Hospital, Jerusalem, at a stock concentration of 4 mg/ml and diluted to 0.1 mg/ml or 0.15 mg/ml in standard mouse acidic drinking water. In the experiments described herein, mice were allowed to choose between fentanyl and quinine-adulterated water (0.1 mg/ml quinine in standard mouse acidic drinking water). Quinine was included to match the intrinsic bitterness of the fentanyl solution.

### Automated home cage hardware

**Fig. 2:**
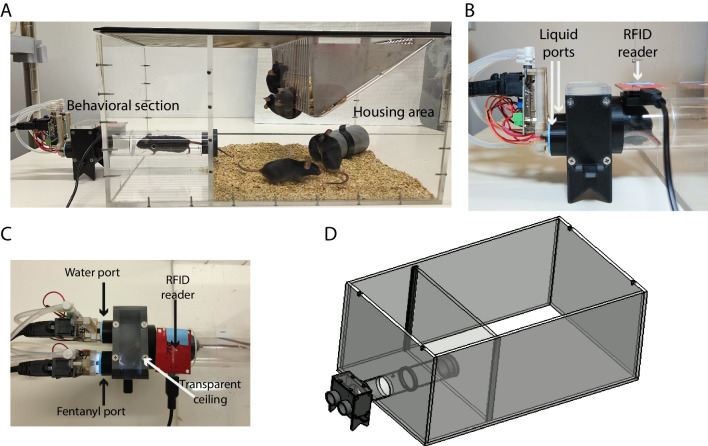
Hardware of the automated self-administration home cage. **A** A photo of a prototypical HOMECAGE setup. The cage is divided into two compartments: a housing area and a behavioral section. **B** A close-up, side-view photo of the behavioral section. A tube leads to the drinking zone where a RFID reader identifies the mouse currently participating, initiating an individually defined trial. When the mouse enters the behavioral section, it can choose from two drinking ports, which record pokes and provide liquid reward. **C** A close-up top-view photo of the behavioral section. The transparent ceiling of the drinking zone allows for video monitoring of the actions of mice. In this implementation, the mice are offered a choice between fentanyl and water, and a light turns on in the fentanyl port when a reward is provided. **D** A schematic diagram of HOMECAGE. Detailed assembly instructions are available online: https://github.com/Citrilab/group_housed_SA.git

The behavioral cage is a Plexiglas structure (size 25.5 × 41.5 × 20.6 cm), divided into two compartments (Fig. 2A): the housing area with bedding, free access to food, and enrichment equipment (as defined by the experimenter), separated from the behavioral ports by a Plexiglas tube, over which the RFID reader (ID Innovations, ID-20LA) is located. We built the cage from 6-mm thick cast Plexiglas sheets, milled with a simple CNC machine or a laser cutter. The cage sheets are affixed to each other with Plexiglas glue and fortified with small screws (see DXF files, https://github.com/Citrilab/group_housed_SA.git). The Plexiglas tube connecting to the ports is of a diameter that only allows for entry of a single mouse at a time (40 mm outer diameter, 36 mm inner diameter) milled by a milling machine. This diameter is suitable for C57BL/6 male mice (> ~ 8 weeks old), when working with younger male mice, female mice, or mice of other backgrounds, adjustments to the tube diameter may be required.

The tube is mounted with a 3D-printed mount on a wall dividing the cage to its two compartments (print file are available online: https://github.com/Citrilab/group_housed_SA.git). The tube leads to a small compartment with two drinking ports, which record the pokes of the individual mice and supply the liquid chosen. The plan allows for easy removal of the tube for cleaning of the cage between mouse cohorts, or as necessary. The behavioral chamber (Fig. 2B, C), consisting of two receptacles for Bpod behavioral ports (Sanworks, Product ID 1009), is 3D printed (print file are available online: https://github.com/Citrilab/group_housed_SA.git) and affixed with a transparent ceiling in order to support the positioning of a camera above the cage. The whole system is controlled by a Bpod state machine (Sanworks, Product ID: 102) that enables control of behavioral experiments, available commercially or as an open source (http://www.sanworks.com). Liquids (fentanyl, water, etc.) are supplied by Bpod behavioral ports (Sanworks, Product ID: 1009); the liquid is contained in disposable syringes and connected to the port via silicone tubes. Detailed assembly instructions, hardware programs, and 3D print files for ports and tube mounts are found online: https://github.com/Citrilab/group_housed_SA.git. Figure 2D provides a schematic illustration of the setup.

### Automated cage software

We used the “Bpod” (Sanworks) software environment (https://github.com/sanworks/Bpod_Gen2) to develop and run our self-administration experiments and added a module of RFID reads to the software. To activate Bpod, the protocol should be written in a state machine syntax. Instruction for general protocol development using Bpod can be found in https://sites.google.com/site/bpoddocumentation/user-guide/protocol-development?authuser=0. Each state in the experiment can trigger actions, while transitions between states are determined by timers, counters, or the actions of subject mice. In our setup, we use the notion of “soft-code” in order to incorporate an RFID reader into Bpod state machine (https://sites.google.com/site/bpoddocumentation/user-guide/function-reference/sendbpodsoftcode?authuser=0).

We took advantage of the state machine structure and dedicated the last state of a trial to activate a soft code byte that repeatedly calls for RFID reads until a new entry is detected. Thus, a new trial is initiated after the identity of the performing mouse is known, allowing the customization of the trial to the individual subject. To assist in the implementation of studies of motivation to consume liquid rewards, we provide working protocols for FR (fixed ratio) and PR (progressive ratio) reinforcement schedules (https://github.com/Citrilab/group_housed_SA.git). In addition, we provide code for a user-friendly GUI (“Bpod helper”) that supports the registration of the subjects and the assignment of individual task parameters.

### Automated self-administration FR (fixed ratio) experiment

**Fig. 3:**
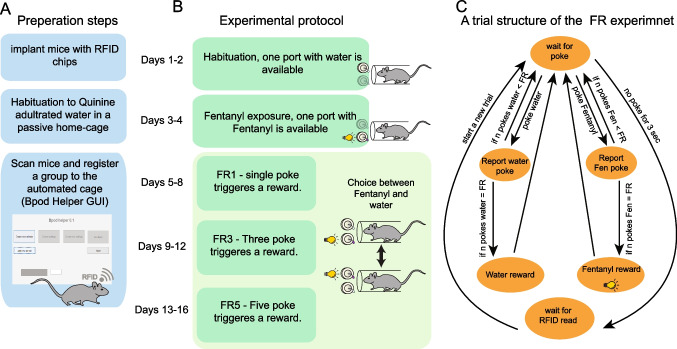
Stages in execution of a group-housed automated self-administration experiment in HOMECAGE. **A** Scheme of the preparation steps prior to initiation of the protocol. Source code for a GUI assisting in the management of the experimental settings for individual mice is provided. **B** Stages in the application of a fixed-ratio (FR) procedure, allowing a choice between orally self-administered fentanyl solution and water. The protocol begins with habituation to each liquid solution separately, then allowing a choice between the two ports, while the number of pokes required for reward delivery (FR) is gradually increased. **C** Trial structure in an FR schedule experiment. A reward is supplied if the FR requirement is performed in a port with an inter-poke-interval of less than 3 s

Mice are first implanted with RFID tags as described in the “surgery” section. Prior to the initiation of an experiment, the cage must be cleaned and dried, and the experimenter must ensure that the ports are functioning properly (detecting pokes and supplying liquid rewards). Then, the size of a liquid drop released by each port must be calibrated, using a tool built into the Bpod package (https://sites.google.com/site/bpoddocumentation/user-guide/general-concepts/liquid-calibration?authuser=0). This step ensures that the drops match in size to avoid biases due to reward size. In our implementation, 2 days prior to entry to the automated cage, we replaced the water in the mice’s regular home cage with quinine-adulterated water (0.1 mg/ml) to habituate the mice to consumption of a bitter liquid (Fig. 3A). Quinine-adulterated water was used in order to balance for the bitterness of fentanyl (Monroe and Radke 2021). After habituation, the mice’s tags were scanned using the “Bpod helper” GUI (“Create new animals” tab), registering each mouse’s name/number in the experiment (Fig. 3A). At the end of the process, the Bpod Helper creates a MATLAB table file with all the names and the tags of the mice in the current cage. Next, the settings of the current stage are determined, using the “Create new settings” tab in the Bpod helper. The settings file includes the liquid drop size and the FR (fixed ratio; the number of pokes required to obtain a reward) for fentanyl or water. In our implementation (Terem et al. 2023), the drop size was set to 10 µl, and the FR requirement was set to 1, then 3, then 5, according to the progression of the experiment. Finally, each mouse is assigned to a defined settings file, using the “choose settings” tab in the Bpod-helper GUI. In our implementation (Terem et al. 2023), all the mice in the cage shared the same settings. However, it is possible to define different settings for each mouse according to experimental design.

Habituation to the experimental cage was achieved by making only one port available during the first 2 days of the experiment, while the holder for the second port was physically blocked, forcing mice to initially consume only water under the FR1 schedule. Trials were initiated by RFID identification of a mouse entering the tube, following which the mouse had a 3-s time window in which to poke at the available port. Following the detection of a poke, a reward was supplied, and the mouse could perform an additional poke.

During the first 2 days, every poke in the available port was rewarded with 10 μl of water (Fig. 3B). A gap of 3 s between pokes or a lack of any poke in the first 3 s of a trial terminated the trial, such that a new RFID read (i.e., an exit and re-entry to the port) was required in order to initiate a new trial (Fig. 3C). The number of trials (entries to the behavioral tube) per mouse was unlimited, as was the number of rewards per trial (if they remained within the 3 s inter-poke-interval). The position of the available port was reversed on the second day of habituation to avoid the development of a side bias. On days 3–4, a single port continued to be active, now containing only fentanyl (0.1 mg/ml), such that each poke was rewarded with 10 µl of fentanyl solution, paired with a light cue, which lit within the port for 500 ms.

As before, the position of the port was flipped on the second day of fentanyl exposure. On subsequent days (day 5 and beyond), both ports were available, and a choice between fentanyl and water was offered (Fig. 3B). Both ports were cleaned daily to avoid blockage of the tube or the sensors, and both ports were tested for functional poke detection and liquid supply. In addition, the identity of the ports (water/fentanyl) was swapped daily to avoid the development of a side bias. After 4 days on the FR1 schedule settings (for both ports), the response requirements for receiving fentanyl reward increased to FR3, such that the 3rd poke was rewarded (for both ports); pokes were accumulated to provide a reward if consecutive pokes were at the same port, in an inter-poke interval of less than 3 s (Fig. 3C). After 4 days in FR3, the response requirement was increased to FR5, and the concentration of fentanyl solution was increased to 0.15 mg\ml (Fig. 3B, C).

### Progressive ratio (PR) experiment

At the end of the FR schedule experiment, some of the mice were tested under the PR schedule where the number of pokes required to receive a reward was increased by 1 every time a reward of the same liquid was provided. All mice started on the FR1 schedule for both fentanyl and water. Following each entry to the tube, the mice poked to obtain a reward, and following reward delivery, the value of the current ratio for the specific reward type and mouse was stored in memory, and a new trial could be initiated. Once a new RFID read was detected, the value of the ratio reached for fentanyl and water for the specific mouse was retrieved, and the number of pokes required to obtain a reward was updated accordingly (code for the protocol is available online: https://github.com/Citrilab/group_housed_SA.git).

## Results

### Hardware and software of the self-administration group-housed home cage

To study fentanyl self-administration in a group-housed setting, we designed and built an automated setup for oral self-administration in the home-cage. The behavioral cage is divided into two compartments (Fig. 2A): a housing area and a behavioral area. The two are separated by a tube leading to the behavioral area, where two drinking ports supply liquid reward and record pokes (Fig. 2B,C). An RFID reader is used for the identification of the individual mouse currently participating (Fig. 2B, C). Detailed instructions for cage assembly are provided online: https://github.com/Citrilab/group_housed_SA.git.

We used the “Bpod” (Sanworks) software environment to develop and run self-administration experiments in the HOMECAGE platform, as described in the methods. We share code for the execution of group-housed fixed ratio (FR) and progressive ratio (PR) schedules in the automated setup (https://github.com/Citrilab/group_housed_SA.git). In addition, we provide code for a user-friendly GUI (“Bpod helper”) that assists with registration of the experimental subjects and assignment of individual task parameters (https://github.com/Citrilab/group_housed_SA.git) (Fig. 3A).

### Fentanyl self-administration in a group-housed home cage

**Fig. 4:**
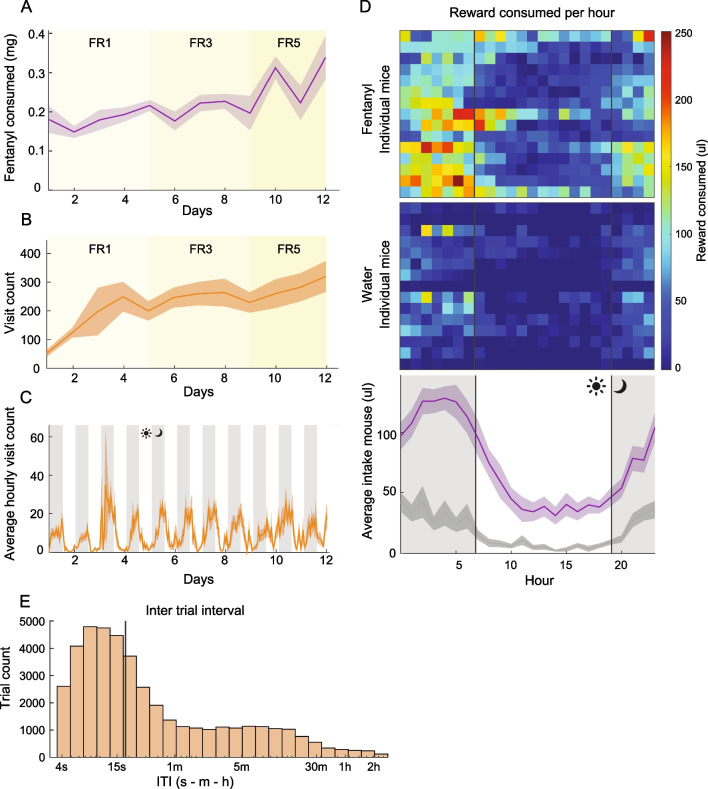
Basic characterization of performance in HOMECAGE. **A** Mice consumed increasing quantities of fentanyl in the oral self-administration automated cage over days. The line represents average consumption per day (mg), while shaded areas represent SEM (from Terem et al. 2023). **B** The line represents the average daily visit count to the behavioral port (RFID reads), while shaded areas represent SEM. **C** Distribution of visits throughout the day in the first 13 days of the FR schedule experiment. The line indicates the average hourly visit count, while the gray-shaded areas represent SEM. The light phase is indicated by a white background, while the dark phase is represented by a gray background. **D** Average liquid consumption per hour of the day. Top: heatmap of average hourly fentanyl intake. Each row represents the performance of an individual mouse. Vertical lines indicate the onset and offset of the light cycle phase. Middle: heatmap of average hourly intake of water during the day. Each row represents the performance of an individual mouse. Bottom: average hourly consumption of fentanyl (purple) vs water (gray) over the course of a 24-h cycle. Shaded gray areas represent SEM. The white background represents the light phase. **E** Distribution of inter-trial intervals, plotted on a logarithmic x-axis scale, illustrating that most trials are clustered with short inter-trial intervals. The vertical line indicates the median of the distribution

**Fig. 5:**
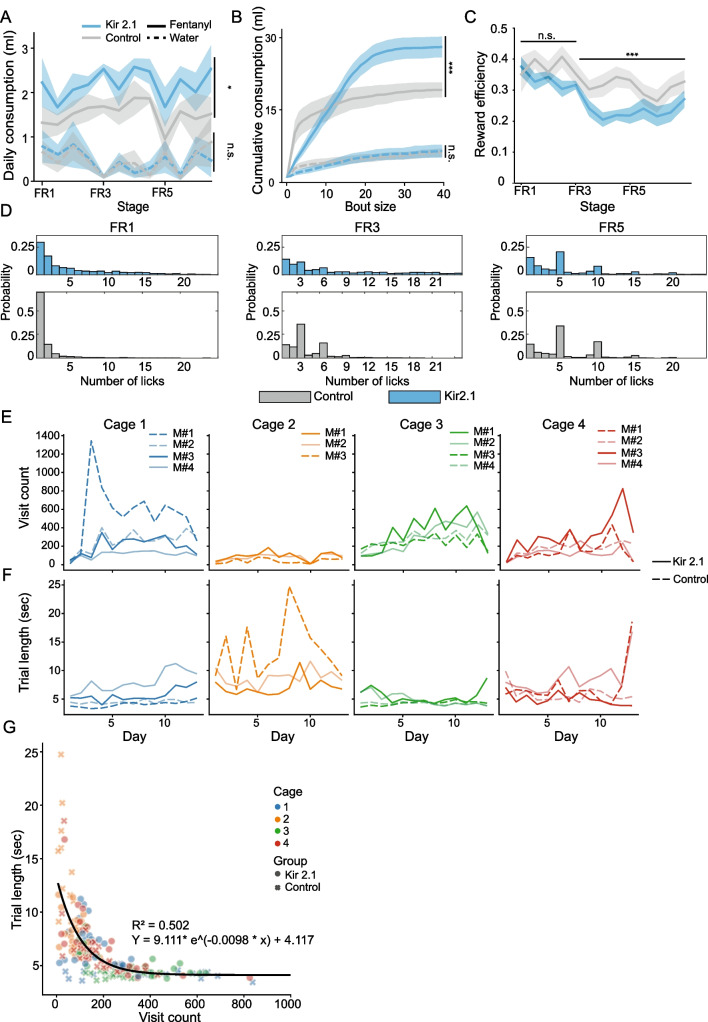
Comparison of the performance of experimentally manipulated mice to co-housed control littermates within the HOMECAGE setup. **A** Mice in which the activity of frontal-projecting claustral neurons was constitutively inhibited by expression of Kir2.1 consumed more fentanyl than control mice while consuming equal amounts of water (from Terem et al. 2023). Lines indicate averages, and shaded areas represent SEM. **B** Claustrum-inhibited mice consumed fentanyl, but not water, in larger bouts (more rewards per visit) than controls. Cumulative distribution of liquid consumption according to bout size (from Terem et al. 2023). **C** Average “reward efficiency” (the probability that a visit to the drinking zone resulted in a reward) for experimental vs control groups. Claustrum-inhibited mice showed reduced reward efficiency at FR3 and FR5 contingencies compared to control mice. **D** Probability distribution of the number of pokes at the fentanyl port per trial (rewarded and non-rewarded pokes). Bars represent the number of trials in which a defined number of pokes was performed, comparing the results for claustrum-deficient mice to control mice. Top—Kir2.1 (claustrum-inhibited) group, bottom—control group (note difference in y-axis scale). Left—FR1 reinforcement schedule; middle—FR3; right—FR5. **E**, **F** Daily visit count **E** and daily average trial length **F** throughout the course of the experiment. Each line represents an individual mouse, while colors indicate different cages. Solid lines depict control mice, and dashed lines depict Kir2.1 mice. Results indicating a “cage-effect,” illustrating the need for within-cage comparison of control and experimental mice. **G** Relation between daily visit count and daily visit duration. Each point represents the behavior of a single mouse on an individual day. Round points depict control mice, *x* depicts Kir2.1 mice, and colors indicate different cages. A decaying exponent was fit to the data points, *R*.^2^ = 0.50. *p<0.05; ***p<0.001

**Fig. 6:**
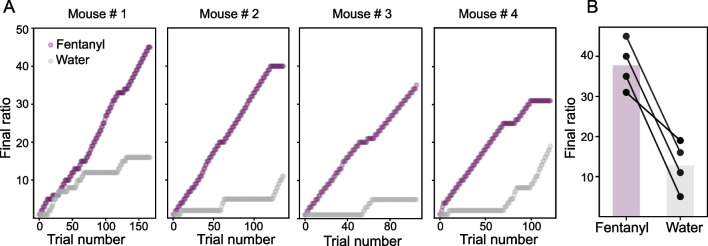
Use of automated individualized parameters in HOMECAGE to perform a progressive ratio schedule in a group-housed setting. **A** Proof of concept for an individualized response on a progressive ratio schedule in a group-housed automated cage. The values of the progressive ratio are updated for individual mice for each reward type throughout the session. **B** Mice perform a higher number of pokes to obtain a fentanyl reward in comparison to water. Lines represent the breaking point for individual mice, while bars represent averages

We describe below the development of fentanyl preference in HOMECAGE, as well as the capabilities of the system and the rich data it can yield. The data described largely stems from experiments aimed at deciphering the role of the claustrum in controlling oral fentanyl self-administration (Terem et al. 2023). However, we currently analyze the behavior of mice more thoroughly to further illustrate the utility of the HOMECAGE method. In the example described herein, 8 mice underwent stereotactic virus injections to establish constitutive inhibition of claustrum neurons projecting to the frontal cortex, while 7 mice served as controls. Experimental and control littermate mice were co-housed (3–4 mice per cage; altogether 4 replicate cages). In the initial analysis of behavior (Fig. 4), we refer to the consumption of all mice regardless of the experimental group, while the impact of experimental manipulation of claustrum activity is described later (Fig. 5). Finally, we provide an additional application of the HOMECAGE system, in the form of the system’s capability to support individualized progressive ratio schedule in group-housed mice (Fig. 6).

In the HOMECAGE system, mice escalated their fentanyl intake over time [Fig. 4A; linear mixed effect model (daily fentanyl ~ day | mouse), *p* < 0.001; adapted from (Terem et al. 2023)]. This is even though the FR requirements increased from FR1, through FR3 to FR5 over time. Furthermore, individual mice stabilized on consistent numbers of visits/day over the days of the experiment, suggesting that mice did not reduce their motivation to interact with the system as the response requirements were increased (Fig. 4B).

Studying the activity pattern of mice in HOMECAGE suggests that the automated system preserves naturalistic features of behavior such as circadian rhythms. Mice tend to enter the behavioral tube more in the dark cycle than in the light cycle and consumed more liquid during the dark cycle (Fig. 4D; linear mixed model (consumption per hour ~ cycle | mouse), *p* < 0.001). Moreover, it is apparent that mice interact with the system in a clustered fashion, as most of the inter-trial-intervals are spaced less than 20 s apart (Fig. 4E). Preliminary experiments performed with co-housed female mice indicate that females exhibit similar dynamics to males (data not shown).

### Inhibition of the claustrum increases fentanyl consumption

We tested fentanyl consumption of mice after inhibition of the activity of frontal-projecting claustral neurons (by expressing Kir2.1 in these projection neurons). Claustrum-inhibited mice consumed more total fentanyl than control cage-mate mice [Fig. 5A; linear mixed model (volume ~ group | mouse), *p* = 0.0206], while drinking equal amounts of water as controls [Fig. 5A; linear mixed model (volume ~ group | mouse), *p* = 0.9924, adapted from (Terem et al. 2023)]. In addition, detailed analysis of the number of rewards per trial revealed that claustrum-inhibited mice consumed fentanyl in larger bouts (more rewards per trial) compared to control mice (Fig. 5B, adapted from (Terem et al. 2023)).

For further investigation of the consumption patterns of the mice, we calculated the daily “reward efficiency” index for each mouse, defined as the probability that a visit in the behavioral area yielded at least one reward. Claustrum-inhibited mice showed lower reward efficiency than control mice at the FR3 and FR5 schedules [Fig. 5C, mixed linear model (efficiency ~ group + FR | mouse), group effect *p* < 0.05, post hoc comparisons with the Bonferroni correction for multiple comparisons comparing claustrum-inhibited to controls per FR schedule: FR1: n.s., FR3: *p* < 0.001; FR5: *p* < 0.001]. Reduced reward efficiency suggests that these mice did not adapt as efficiently as control mice to the new task requirements (an increase in the FR requirements). Interestingly, reduced reward efficiency in the experimental group did not result in reduced fentanyl intake, but rather experimental mice consumed more fentanyl than did mice from the control group (Fig. 5A, C).

Analysis of the distribution of pokes in the fentanyl port per trial (all pokes: rewarded as well as non-rewarded) showed a difference in the pattern of poking between the two groups (Fig. 5D; KS test for the distribution of fentanyl poke number per trial of claustrum inhibited vs control per FR schedule: FR1: *p* < 0.0001, FR3: *p* < 0.0001, FR5: *p* < 0.0001). Under the FR1 contingency, claustrum-inhibited mice had fewer trials in which they obtained a single reward compared with controls but rather obtained their increased consumption of fentanyl by performing larger bouts, obtaining multiple rewards. Under the FR3 contingency, while control mice appeared to entrain their behavior to the contingency, preferentially performing pokes in multiples of 3, claustrum-deficient mice continued to engage in trials of longer bouts, exhibiting reduced entrainment to the reinforcement schedule (Fig. 5D). The same effect can be seen in the FR5 contingency, where a histogram of poke number per trial shows that control mice exhibit peak numbers of pokes that correspond to the rewarded poke numbers (5, 10, 15), while the distribution of pokes for the claustrum-inhibited mice is shifted to a larger number of rewards per trial and was less tightly associated with fentanyl consumption (Fig. 5D).

This difference in the distribution of pokes around rewarded or non-rewarded pokes points not only at the tendency of claustrum-deficient mice to perform longer bouts but also shows that claustrum-inhibited mice exhibit a deficit in optimizing their performance according to changing task parameters, as they perform a higher proportion of unrewarded pokes, resulting in more failed trials than the control mice (Fig. 5C).

Together, there are two potential explanations (which are not mutually exclusive) for the behavioral changes of the claustrum-inhibited mice in our study: (A) Activity of frontal-projecting claustrum neurons is important for adjustment to modifications of task contingencies and/or (B) Reward efficiency is reduced in claustrum-deficient mice as a consequence of the pharmacological impact of increased fentanyl intake. Experiments designed to support or refute these two possible explanations merit future investigation.

### Mice exhibit persistent individual behavioral patterns, necessitating within-cage controls

The “HOMECAGE” system provides detailed data corresponding to the performance of mice, enabling extensive characterization of individual behavior. Addressing differences in the interaction of individuals with the drinking ports, we calculated the average daily visit count of each mouse, and the average trial length, with longer trials indicating a larger number of pokes per trial. Plotting these two values for each mouse, we observed that mice who perform higher daily visit counts tend to perform shorter trials, and vice-versa, mice who perform longer visit durations tend to visit the behavioral area less frequently (Fig. 5G).

An exponential decay fit for the daily visit duration vs. the daily visit count explains 50% of the variance (Fig. 5G). As described in the “Methods” section, the experiment was comprised of 4 replicate cages, each containing mice from both the control and experimental groups. When plotting the dynamics of visit count and visit length as a function of time, we observed that mice appear to maintain a consistent behavioral profile during the experiment (Fig. 5E, F).

Furthermore, the behavioral analyses indicate that each cage has its unique behavioral “profile,” with noticeable differences between cages both in visit count (Fig. 5E) and visit duration (Fig. 5F). These differences between cages emphasize the importance of co-housing experimental and control groups in the same cage to reduce experimental confounds. Potentially, the persistent difference in performance within a cage may relate to social hierarchy, whereby dominant mice control the availability of the port for their subordinate peers. As the data obtained with the HOMECAGE setup provides detailed information regarding the identity of the visiting mouse and their port visits’ schedule, the system can be potentially used, while implementing further analyses, to study social hierarchy in group-housed mice who consume fentanyl.

### Mice display higher motivation to receive fentanyl reward than water reward

A common procedure to study the motivation to self-administer an addictive drug is the progressive ratio (PR) schedule. Traditionally, PR assessment has been assessed in single-housed laboratory animals, since the number of pokes required for a reward in the current trial is defined by trial history, requiring the tracking of the history of performance of each subject (Richardson and Roberts 1996; Stafford et al. 1998). The implementation of RFID identification in HOMECAGE allows the updating of the current ratio requirement reached by each mice, activating the appropriate next schedule requirement. As a proof of concept for the capacity of HOMECAGE to automatically adapt individualized task parameters based on the mouse’s recent history of performance, we implemented a progressive ratio procedure in our system. Every time a mouse received a reward of either fentanyl or water, the number of pokes required to receive the same type of reward in the next trial increased by one. All tested mice performed more pokes to receive fentanyl than they did to receive water, indicating higher motivation for consumption of fentanyl (individual data are presented in Fig. 6, paired *t*-test for the final ratio reached for fentanyl vs. water, *p* = 0.01).

## Discussion

We established HOMECAGE (Home-cage Observation and Measurement for Experimental Control and Analysis in a Group-housed Environment), a procedure to study group-housed oral fentanyl self-administration. In the HOMECAGE setup, mice preserve their naturalistic social interactions and activity rhythms. Using this procedure, we showed that mice volitionally chose to consume fentanyl over water and escalated their fentanyl consumption over time. In addition, the mice performed more pokes to receive fentanyl than water reward (Fig. 6). We used this system to compare between two co-housed experimental groups (claustrum-inhibited vs controls) (Fig. 5) and observed that claustrum-inhibited mice consumed more fentanyl than controls and consumed the drug preferentially in larger bouts (Terem et al. 2023). We also characterized individual and group differences in consumption patterns, including the number of pokes per trial, trial duration, and trial count (Fig. 5); all of which are important behavioral parameters of drug self-administration.

Our system allows for self-administration of all mice in a group, co-housed, in a way that more closely mimics the real-life experience of human who use addictive drugs. This is enabled by the automation of the system, allowing mice to perform according to their circadian rhythm and not depending on the schedule of the experimenter. This relatively naturalistic setup allows for innate social interactions and minimizes experimenter interference (de Abreu and Kalueff 2021; Neff 2018). Currently, available models of drug consumption or self-administration typically house mice in individual cages or operant chambers. At best, rodents are housed in adjacent cages that are separated by a mesh, in what is still a relatively non-naturalistic setting (Fulenwider et al. 2020; Peitz et al. 2013; Ródenas-González et al. 2023; Slivicki et al. 2023; Smith and Pitts 2014; Thanos et al. 2007; Venniro et al. 2022).

As individuals suffering from opioid addiction may exhibit social withdrawal and often consume opioid drugs in isolation (Badiani 2013; Badiani et al. 2011), the choice of the experimental conditions (isolated vs group-housed) should reflect an informed decision by the investigators regarding the specific aspects of drug consumption and addiction they intend to model. In this regard, HOMECAGE can also serve for experimentation in socially isolated mice, and simple adaptations to the system can allow mice to choose between social isolation and group housing while monitoring their opioid consumption under either condition.

The group-housing in our setup does not come at the expense of data precision, since our system allows not only for the tracking of the behavior of individual mice but can also generate individually tailored task parameters within a single homecage based on the experimenter’s choice or the performance history of the mouse (Fig. 6). A key feature of our procedure is that it allowed for oral opioid self-administration in the mice’s home environment, the preferred environment for opioid self-administration in both rodents and humans (Badiani 2013; Caprioli et al. 2007, 2009).

A key aspect in the investigation of drug self-administration is the element of choice (Banks and Negus 2017; Heilig et al. 2016; Townsend et al. 2021). HOMECAGE offers a methodological approach to assess drug preference through direct comparative assessment of consumption of different drug concentrations, as well as the resilience to adulteration of the drug with a bitter substance (quinine) (Terem et al. 2023). HOMECAGE further supports the expansion of the repertoire of procedures an experimenter could use by flexible modulation of the number of active liquid supply ports, or sensory stimuli. The assessment of drug preference is a valuable tool for understanding the addictive potential of drugs (Johanson and Fischman 1989; Nader et al. 1993; Nader and Banks 2014). Thus, the HOMECAGE method can be used to assess the abuse liability of opioid drugs at various concentrations, to evaluate the effect of manipulations or treatments on drug preference, and to compare the opioid preference in mouse models of various psychiatric disorders. Finally, it is worth noting that other approaches are also being developed for monitoring individualized oral self-administration in socially housed rodents, such as PiDose (Woodard et al. 2020) for mice and FARESHARE (Frie and Khokhar 2023) for rats.

HOMECAGE yields rich and detailed data that can be used to analyze not only the amount of fentanyl consumed or the effort the mouse is willing to make to consume oral fentanyl but also the pattern of its consumption, such as the duration of bout intake, the inter-trial intervals between volitional drug choices, circadian dynamics of consumption, and the distribution of number of pokes (rewarded and non-rewarded) per trial. Interested users are likely to identify additional meaningful patterns in the rich data provided by the system, such as insights into the social hierarchy and dynamics between co-housed mice. Future work could further characterize and optimize the experimental conditions of fentanyl and quinine concentrations.

## Potential implementations for further research

The proposed automated group-housed oral fentanyl self-administration system complements classical assays of drug reward and choice in animal models (Banks and Negus 2017; Sanchis-Segura and Spanagel 2006; Shaham et al. 2003; Venniro et al. 2020). For example, a methodological assessment of dose–response curves for different fentanyl concentrations is enabled by controlling the concentration of the fentanyl solution or the drop size per reward. Similarly, one could study the willingness of mice to continue and consume fentanyl despite negative consequence by adulterating the fentanyl solution with escalating concentrations of quinine (or including an electrified barrier in the setup). A conditioned stimulus can be added to the fentanyl delivery and can be used for extinction and reinstatement procedures (Shaham et al. 2003) to measure relapse-related behaviors. Using the individually assigned task parameters, one can tailor abstinence and relapse models (Fredriksson et al. 2021) to the individuals participating in the experiment by controlling whether the different ports are active according to the history of each experimental subject.

Finally, the HOMECAGE procedure will enable to conduct further studies relevant to drug addiction, ranging from evaluating the abuse liability of investigative new drugs to comparing different pharmacological and non-pharmacological approaches for decreasing addiction-related behaviors. HOMECAGE also enables the development of new experimental procedures, for example, studying co-housed groups of peers or familial structures, in which some participants have access to the opioid drug, while others do not. This will allow assessment of the consequences of opioid consumption on individuals embedded within their natural social network, as well as the protective/harmful nature of different social interactions on opioid consumption. The HOMECAGE procedure can also be used to study the trans-generational impact of drug consumption.
