# Correction to: An automated group‑housed oral fentanyl self‑administration method in mice

**DOI:** 10.1007/s00213-024-06547-3

**Published:** 2024-01-30

**Authors:** Noa Peretz‑Rivlin, Idit Marsh‑Yvgi, Yonatan Fatal, Anna Terem, Hagit Turm, Yavin Shaham, Ami Citri

**Affiliations:** 1 Edmond and Lily Safra Center for Brain Sciences, The Hebrew University of Jerusalem, Edmond J. Safra Campus, Givat Ram, 91904 Jerusalem, Israel; 2 Institute of Life Sciences, Hebrew University of Jerusalem, Edmond J. Safra Campus, Givat Ram, 91904 Jerusalem, Israel; 3 Behavioral Neuroscience Branch, IRP/NIDA/NIH, Baltimore, MD USA; 4 Program in Child and Brain Development, MaRS Centre, West Tower, Canadian Institute for Advanced Research, 661 University Ave, Suite 505, Toronto, ON M5G 1M1 Canada

**Correction to: Psychopharmacology**

10.1007/s00213-024-06528-6

In the published paper, the label of the Y-axis of figure 5C has been distorted, this should read as “Reward Efficiency”. In the Funding section, there is a placeholder for the designation of the institution that supported open access, and this has not been updated to designate “The Hebrew University of Jerusalem”.

The original article has been corrected.

